# Next-generation antibody–drug conjugates revolutionize the precise classification and treatment of HER2-expressing breast cancer

**DOI:** 10.20892/j.issn.2095-3941.2023.0286

**Published:** 2023-10-12

**Authors:** Lei-Jie Dai, Yu-Wei Li, Ding Ma, Zhi-Ming Shao, Yi-Zhou Jiang

**Affiliations:** 1 Department of Breast Surgery, Fudan University Shanghai Cancer Center, Shanghai 200032, China; 2 Key Laboratory of Breast Cancer in Shanghai, Fudan University Shanghai Cancer Center, Shanghai 200032, China

The concept of antibody–drug conjugations (ADCs) can be tracked back to the early 20^th^ century when the renowned immunologist, Paul Ehrlich, proposed the idea of a “magic bullet”, which utilizes ADCs for targeted destruction of microorganisms and tumor cells^1^. After nearly one century of development, ADCs have emerged as a rather promising approach in the treatment of cancer, especially breast cancer, which is the most common malignant tumor in women^2^.

Human epidermal growth factor receptor 2 (*HER2*), also known as Erb-B2 receptor tyrosine kinase 2 (*ERBB2*), is a crucial molecular classifier for breast cancer. The *HER2* status is determined using immunohistochemistry (IHC) and *in situ* hybridization (ISH) in pathology laboratories. Approximately 15% of breast cancer patients exhibit HER2 overexpression and are thus defined as HER2-positive breast cancers. HER2 was a pivotal driver and therapeutic target in these patients^3^. HER2 is not considered to be a driving factor and is conventionally not targetable for breast cancers that display low-to-moderate HER2 expression (usually referred to as HER2-low breast cancers)^4^.

The emergence of next-generation ADCs, represented by trastuzumab deruxtecan [T-DXd (also called DS-8201)], has revolutionized the precise classification and treatment of HER2-expressing breast cancer, including both HER2-overexpressing HER2-positive breast cancers and HER2-moderately expressed HER2-low breast cancers. By summarizing the unique mechanism of next-generation ADCs and their application in HER2-expressing breast cancers, we hope to examine the great impact of next-generation ADCs on clinicians and researchers, and to stimulate more emphasis on adapting to the era of next-generation ADCs.

## Unique mechanism of next-generation ADCs

ADCs are comprised of a monoclonal antibody, a linker, and a cytotoxic payload. This unique design enables ADCs to achieve “targeted chemotherapy” and provide advantages over conventional chemotherapy and targeted therapies^5^. Chemotherapy often lacks tumor specificity, exhibits dose-dependent toxicity, and has a narrow therapeutic window. Conventional targeted therapies necessitate the presence of specific molecular targets (e.g., overexpressed HER2), which restricts applicability to a relatively limited population. In contrast, ADCs circumvent these constraints by providing targeted delivery of cytotoxic agents guided by specific antibodies, which reduces damage to healthy cells and enhances tumor cell destruction^5^.

**Figure 1:**
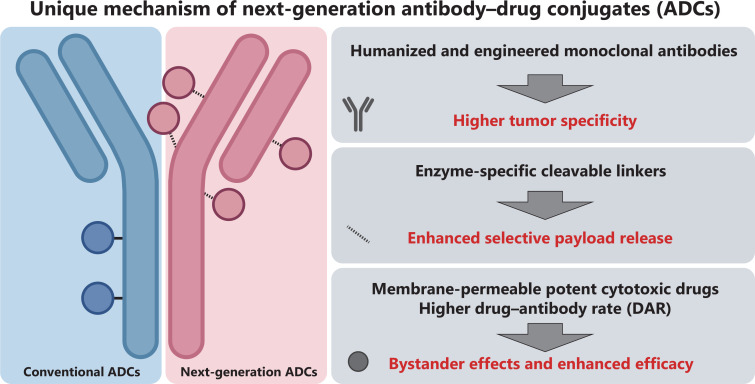
Schematic of the unique mechanism of next-generation antibody‒drug conjugates (ADCs).

ADCs have undergone remarkable advancements in recent years. Next-generation ADCs (or 3^rd^ generation ADCs) have made substantial progress in antibody optimization, linker innovation, and payload selection (**Figure 1**). Humanized and engineered monoclonal antibodies increase tumor specificity, cleavable linkers enhance selective payload release, and membrane-permeable potent cytotoxic drugs contribute to bystander effects and enhanced efficacy. These advances have expanded the scope of ADC application, improved therapeutic efficacy, and reduced the incidence of adverse events^6^. Currently, there are many next-generation ADCs on the market or in development for breast cancer, such as T-DXd, SHR-A1811, and RC-48. Comprehensive summaries of these next-generation ADCs have been provided by previous studies^7,8^.

## Next-generation ADC reshapes the treatment landscape for advanced HER2-positive breast cancer

The prognosis of advanced HER2-positive breast cancer patients has been improved significantly in the past two decades^9^. The underlying reason can be attributed to the continuous development of anti-HER2 targeted therapies, such as the monoclonal antibody, pertuzumab, and various small molecule compound tyrosine receptor inhibitors (TKIs)^3^. Therefore, we have entered a new era of next-generation ADCs.

In the DESTINY-Breast03 trial, advanced HER2-positive breast cancer patients treated with the next-generation ADC, T-DXd, achieved a > 4-fold increase in the median progression-free survival (PFS) compared to T-DM1, reaching a median PFS of 28.8 months^10^. This significant positive result has made T-DXd the only priority for second-line treatment of advanced HER2-positive breast cancer, substituting for the previous standard treatment (T-DM1) in the 2023 NCCN guidelines^11^.

Currently, more than 10 ADCs, most of which are next-generation ADCs, are undergoing clinical trials with the aim to become one of the choices for second-line treatment for advanced HER2-positive breast cancer^8^. Indeed, significant growth and competition for next-generation ADCs may be observed in the foreseeable future.

## Value of next-generation ADCs in the treatment of HER2-low breast cancer

Compared to the significant improvement in survival reported in HER2-positive breast cancer, the survival benefits for HER2-negative breast cancer patients have been relatively limited in the past 20 years^9^. The development of next-generation ADCs provides opportunities for a subset of HER2-negative breast cancers, which are now commonly designated HER2-low breast cancers, that have low-to-moderate HER2 expression.

HER2-low status is defined as HER2 IHC 1+ or 2+ with ISH-. Based on this definition, HER2-low breast cancer is a prevalent subtype, accounting for a significant proportion (∼50%) of cases^12^. The phase Ib DS8201-A-J101 trial^13^ and phase II DAISY trial^14^ confirmed the efficacy and safety of the next-generation ADC, T-DXd, in advanced HER2-low breast cancers. Then, the randomized control phase III DESTINY-Breast04 trial^15^ further confirmed the impressive survival benefit of T-DXd treatment for advanced HER2-low breast cancer over chemotherapy, which made T-DXd the preferred treatment for advanced HER2-low breast cancer in the second-line setting for HR-positive and -negative diseases^11^.

The success of T-DXd brought a new therapeutic choice for these HER2-negative breast cancers, for which moderately expressed HER2 was previously not targetable. There are some potential competitors for T-DXd that are undergoing phase I and II clinical trials in advanced HER2-low breast cancers^12^. Notably, for advanced HER2-low breast cancers, the Trop2-targeted next-generation ADC, sacituzumab govitecan (SG), has also been verified to be the preferred treatment in the second-line setting^11^.

## Perspectives on the precision management of HER2-expressing breast cancer in the era of next-generation ADCs

The application of T-DXd in advanced HER2-positive and -low breast cancers showed the strong potential of next-generation ADCs and raises crucial problems for future clinical trials and translational studies.

### Expanding the beneficiary population of ADCs

The scope of next-generation ADCs represented by T-DXd is continuously broadening. The applicable population of ADCs has extended from patients who had a treatment failure in multiple lines of therapy in the second- or first-line setting and holds potential for early-stage breast cancer. This extension relies on the progression of high-quality randomized controlled trials to provide robust evidence, which are now underway. The DESTINY-Breast11 study explored the effectiveness of T-DXd as neoadjuvant treatment for HER2-positive breast cancer^16^. The TALENT study investigated the efficacy of combining T-DXd with endocrine therapy in neoadjuvant treatment for HR+/HER2-low early-stage breast cancer^17^. The DESTINY-Breast05 study included high-risk HER2+ breast cancer patients who did not achieve pCR after neoadjuvant treatment. The patients were randomized to receive T-DXd or T-DM1 adjuvant therapy to determine the efficacy of the two ADCs^18^. Next-generation ADCs are being used in an attempt to expand and influence the landscape of early-stage breast cancer treatment, holding tremendous potential for offering patients more treatment choices and hope. Efforts are also made to extend the lower limit of the HER2 level for next-generation ADC use. The results of the DAISY trial suggested the existence of T-DXd responders in HER2-0 tumors^14^, which exhibited even lower HER2 expression than HER2-low tumors. Thus, defining an accurate lower limit for the HER2 level in the application of T-DXd is urgently needed.

### Exploring the layout of ADCs and other therapies

Another aspect to consider is how next-generation ADCs can be strategically positioned alongside existing therapies. Breast cancer treatment benefits from a wide array of available therapeutic agents. It is essential to integrate ADCs into the existing treatment regimens, especially for hormone receptor-positive breast cancers, for which there is a rich selection of mature therapies and a relatively good prognosis. Clinical trials have actively explored the combination of T-DXd with existing therapies^19^; however, additional supporting data are needed. Another vital issue is how to treat patients who develop resistance to next-generation ADCs. The mechanisms underlying ADC resistance primarily involve three factors: antibody binding; payload; and internalizing and trafficking processes^20^. There are currently two main strategies to address ADC resistance: the development of new drugs; and the use of combination therapies^21^. Dual-site targeting with HER2 antibodies and coupling with novel toxins may offer promising prospects for overcoming resistance in the course of novel drug development^22^. Furthermore, some studies have explored the combination of ADCs with other therapies to combat resistance, such as ADCs in combination with pertuzumab, TKIs, or immunotherapy^23,24^. Resistance is indeed inevitable, but the unique structure and mechanism underlying ADC action provide avenues for addressing resistance, thus prospective clinical trials are needed.

### Promoting the diagnostic accuracy of HER2-low status

Accurate pathologic diagnosis of HER2-low is crucial. Several studies have shown that the current diagnostic methods for HER2-low based on IHC and ISH are not entirely robust. Specifically, a multicenter study published in *JAMA Oncology*^25^ reported that a panel of pathologists had a concordance rate of only 26% in their interpretations of HER2 IHC 0 and IHC 1+. Recent years have witnessed the potential of combining digital pathology and artificial intelligence (AI) models. The development of a more accurate method to accurately diagnose HER2-low tumors might also benefit from this trend, which allows for minimization of interobserver discrepancies^26^. Other studies are also trying to establish diagnostic pipelines beyond the current system based on IHC and ISH to determine HER2-low status more quantitively and reproducibly^27^.

### Seeking more precise effect predictors of ADCs

Researchers are utilizing various cohorts, especially cohorts from clinical trials, to identify biomarkers for accurate efficacy prediction. The exploration of predictive markers for T-DXd efficacy extended beyond traditional clinical and pathologic indicators in the DAISY trial. By examining digital images of hematoxylin-eosin staining slides with the AI model, researchers found that a baseline spatial feature may predict a worse response, which is characterized by the presence of large areas of HER2 IHC 0 cells distinct from HER2 IHC 3+ tumor cells^14^. Based on these potential biomarkers, feasible and reliable diagnostic tools can be developed and further validated in prospective clinical trials.

### Overcoming primary and secondary drug resistance

Investigating the mechanism of primary and secondary drug resistance and how to overcome resistance is a high priority with the increased use of ADCs. By comparing molecular features between responder and non-responder clinical trial cohorts and paired samples before and after treatment, it is possible to effectively identify the underlying mechanisms and overcoming strategies. Notably, leveraging preclinical models, such as patient-derived organoids (PDOs), can recapitulate the molecular characteristics and treatment sensitivity of original patient tumors^28^. These features allow for high-throughput screening of potential strategies to reverse drug resistance and help accelerate the clinical application of translational research.

### Investigating ethnic differences in HER2-low breast cancer

There may be differences in drug efficacy among different ethnicities. In the subgroup analysis of the DESTINY-Breast04 trial, Asian patients with HER2-low breast cancer may be more likely to benefit from T-DXd than patients of other ethnicities in the HR-negative subgroup^15^. Additional study is warranted to clarify the role of ethnicity in HER2-low breast cancer because these factors may ultimately impact the efficacy of therapeutic interventions.

## Next-generation ADCs redefine treatment strategies and research approaches for breast cancer

The DESTINY-Breast trials are among the best-known clinical trials in the study of next-generation ADCs. The DESTINY-Breast trials proved the impressive effectiveness of T-DXd in breast cancer and provided a new therapeutic choice for patients. The DESTINY-Breast trials, however, have also challenged conventional concepts and persuading clinicians and researchers to establish new knowledge frameworks.

### Clinical practices are revolutionized by new therapeutic strategies

The emerging ADCs are gradually changing the treatment landscape of advanced breast cancer in the second-line settings and beyond for HER2-positive breast cancers, and are poised to be adopted as front-line treatments. The status of the previous generation ADC, T-DM1, is being challenged. Although T-DM1 is still the standard treatment for adjuvant therapy of high-risk non-pCR patients after neoadjuvant therapy, especially for early-stage breast cancers, the ongoing DESTINY-Breast05 trial aims to rewrite the guidelines^18^. The clinical status of T-DM1 might be repositioned with the process of DESTINY-Breast series trials. ADCs have opened up an entirely new chapter for the treatment of patients with HER2-low breast cancers, which account for approximately 50% of all metastatic breast cancer cases and were not previously intended for HER2-targeting treatments.

### The 2-tier classification of HER2 status might be modified

The 2-tier HER2-positive/-negative classification has guided the treatment of breast cancer for two decades, with HER2-positive tumors allotted to receive HER2-targeting therapies and HER2-negative tumors to receive other medications. The boundary between HER2-positive and -negative cells in clinical practice seems to be blurred, however, by the successful application of HER2-targeting next-generation ADCs in HER2-low breast cancers. Although it has been commonly acknowledged that HER2-low breast cancer is not a distinct molecular entity, the current 2-tier classification is not sufficient to guide the application of next-generation ADCs in breast cancer. Given the broader application of next-generation ADCs, a modified classification of HER2 status beyond the 2-tier classification might need to be established by introducing new quantitative methods or spatial features.

### Research approaches to studying breast cancer heterogeneity might be redirected

First, the classic data-driven analysis method might turn to clinical outcome-driven analyses to study the heterogeneity of breast cancers. In data-driven studies, researchers usually employ unsupervised clustering methods regardless of clinical outcomes. Classifications have recently emerged, including clinical outcomes, especially for those outcomes based on cohorts derived from clinical trials, such as ISPY-2^29^. This trend suggests that integrating precise classification and treatment outcomes might contribute to more accurate and feasible methods to guide bedside practice. Second, transformation might also occur in the development of novel drugs. In the past, researchers and drug developers have placed more emphasis on searching for new targets, elucidating the underlying mechanisms, and ultimately finding corresponding targeting compounds. With the maturation of ADC technology and the success of T-DXd, drug enterprises may turn to utilizing existing classic targets, such as HER2, HER3, and EGFR, to develop more efficient ADCs^6^.

Both challenges and opportunities exist in the era of next-generation ADCs. For drug enterprisers, the success of next-generation ADCs have challenged the status of former standard treatments, but also strongly proved the feasibility of ADC technology and opened a new track of drug development. For clinicians, next-generation ADCs added to the toolbox of treatment and provided new opportunities for patients, especially patients who were heavily pretreated. Next-generation ADCs also put advanced vital issues, such as toxicity management. Next-generation ADCs incentivized fundamental researchers to adapt to reshaped research approaches and also offered new topics, such as studying how tumors develop resistance to ADCs and how to treat tumors after ADC resistance. In summary, the emergence of next-generation ADCs has significantly changed the landscape of breast cancer treatment and research. Ongoing research, coupled with regulatory approvals, underscores the promising future of ADCs in combating breast cancer. With continued advancements, ADCs hold the potential to provide more precise and efficacious treatment options, ultimately improving patient survival and quality of life.
